# An update of Nrf2 activators and inhibitors in cancer prevention/promotion

**DOI:** 10.1186/s12964-022-00906-3

**Published:** 2022-06-30

**Authors:** Farhad Pouremamali, Amir Pouremamali, Mehdi Dadashpour, Narges Soozangar, Farhad Jeddi

**Affiliations:** 1 Department of Medical Biotechnology, Faculty of Advanced Medical Sciences, Tabriz University of Medical Sciences, Tabriz, Iran; 2 Department of Virology, Faculty of Medical Sciences, Tarbiat Modares University, Tehran, Iran; 3 Department of Medical Biotechnology, Faculty of Medicine, Semnan University of Medical Sciences, Semnan, Iran; 4 Cancer Research Center, Semnan University of Medical Sciences, Semnan, Iran; 5 Digestive Diseases Research Center, Ardabil University of Medical Sciences, Ardabil, Iran; 6 Zoonoses Research Center, Ardabil University of Medical Sciences, Ardabil, Iran; 7 Department of Genetics and Pathology, School of Medicine, Ardabil University of Medical Sciences, Ardabil, Iran

**Keywords:** Nrf2 activators, Cancer, Nrf2 inhibitors, Keap1

## Abstract

NF-E2-related factor 2 (Nrf2) protein is a basic-region leucine zipper transcription factor that defends against endogenous or exogenous stressors. By inducing several cytoprotective and detoxifying gene expressions, Nrf2 can increase the sensitivity of the cells to oxidants and electrophiles. Transient Nrf2 activation, by its specific activators, has protective roles against carcinogenesis and cancer development. However, permanent activation of Nrf2 promotes various cancer properties, comprising malignant progression, chemo/radio resistance, and poor patient prognosis. Taken together, these findings suggest that reaching an optimal balance between paradoxical functions of Nrf2 in malignancy may render a selective improvement to identify therapeutic strategies in cancer treatment. In this review, we describe lately discovered Nrf2 inducers and inhibitors, and their chemopreventive and/or anticancer activities.

**Supplementary Information:**

The online version contains supplementary material available at 10.1186/s12964-022-00906-3.

## Plain english summary

The Nrf2 pathway signifies one of the most significant cell defense procedures against exogenous or endogenous stressors. Certainly, by increasing the expression of several cytoprotective genes, the transcription factor Nrf2 can shelter cells and tissues from multiple sources of damage including electrophilic, xenobiotic, metabolic, and oxidative stress. Notably, the aberrant activation or accumulation of Nrf2, a common event in many tumors, confers a selective advantage to cancer cells and is connected to malignant progression, therapy resistance, and poor prognosis. Therefore, lately, Nrf2 has arisen as a hopeful target in treatment of cancer, and many struggles have been made to detect therapeutic strategies intended at disrupting its pro-oncogenic role. By summarizing the outcomes from past and recent studies, this review provided an overview concerning the Nrf2 pathway and the molecular mechanisms causing Nrf2 hyperactivation in cancer cells. Finally, this paper also described some of the most promising therapeutic approaches that have been successfully employed to counteract Nrf2 activity in tumors, with a particular emphasis on the development of natural compounds and the adoption of drug repurposing strategies.

**Video abstract**

**Supplementary Information:**

The online version contains supplementary material available at 10.1186/s12964-022-00906-3.

## Background

Despite the numerous efforts that researchers have carried out to improve the cancer outcomes, the overall cancer mortality rate has not significantly diminished over the past 30 years [1–3]. Reactive oxygen species (ROS) and reactive nitrogen species (RNS) are produced in the cells as the result of normal physiological procedures such as during inflammatory responses and mitochondrial aerobic respiration [4]. The elevated amounts of ROS and RNS in cancer cells can be responsible for triggering oxidative stress, which leads to DNA damage, alterations in tumor-suppressor genes and eventually initiation, development, and progression of cancer [4–6]. One of the key systems that confronts oxidative stress and xenobiotics is the Nrf2 signaling system.

**Fig. 1:**
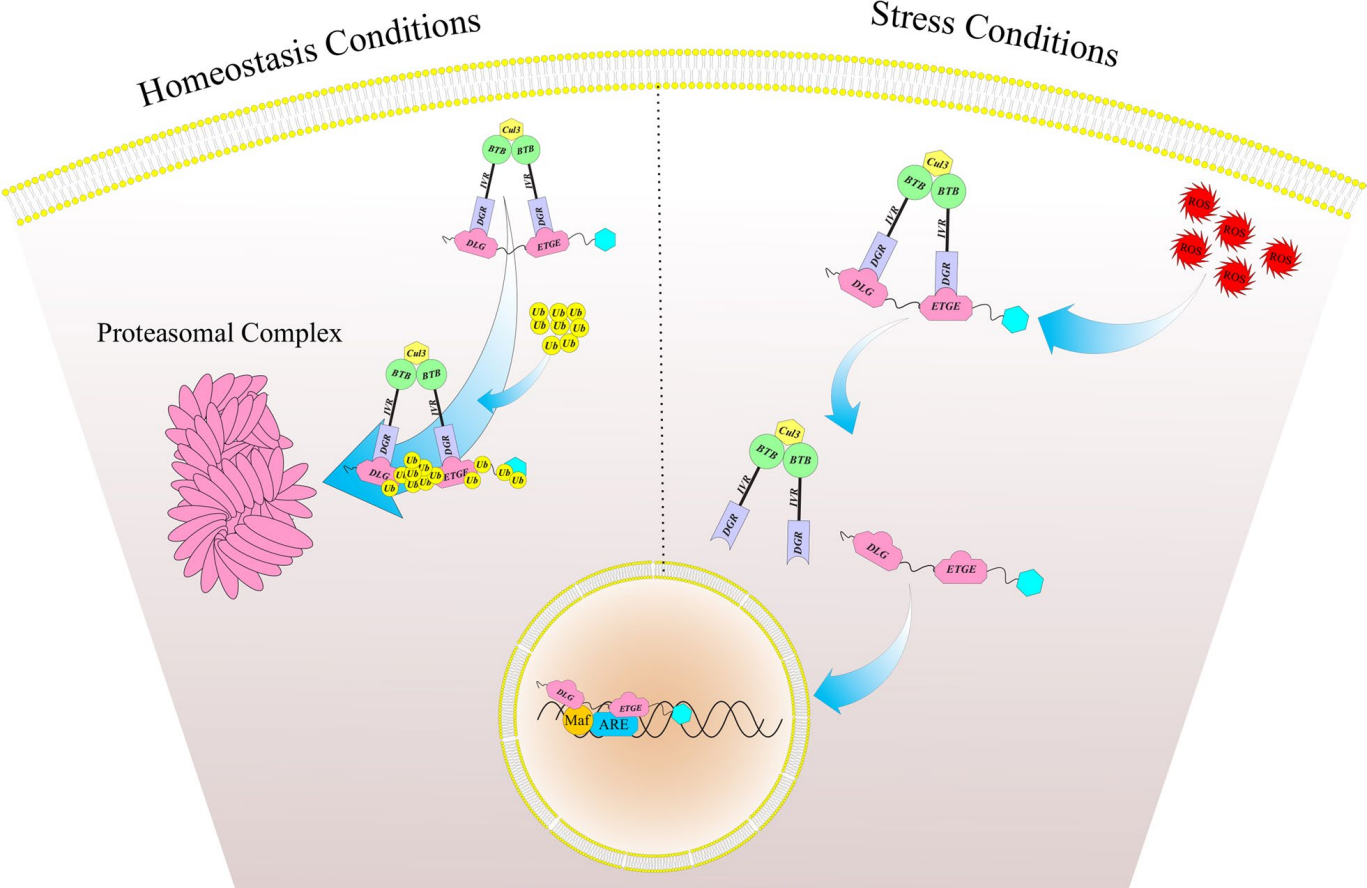
Nrf2 pathway under homeostasis and stress conditions. Under unstressed situations, most of Nrf2 protein is ubiquitinated by Keap1-Cullin3 E3 ubiquitin ligase enzymes and subsequently degraded by the proteasomal machinery. Upon exposure to oxidative stresses, the interaction between Nrf2 and that complex is interrupted, which resulted in decreased Nrf2 degradation and increased its translocation into the nucleus where heterodimerized with the small MAF proteins

Nuclear factor erythroid 2 [NF-E2]–related factor 2 (also called NFE2L2 or Nrf2) is a basic-region leucine zipper (bZIP) transcription protein and a member of CNC (cap ‘‘n’’ collar) family chiefly localized in the cytoplasm [7]. Kelch-like ECH-associated protein 1 (Keap1) or inhibitor of Nrf2 (INrf2) is a redox-regulated substrate adaptor protein for a Cullin-3/Rbx-1 ubiquitin ligase complex [8]. Under unstressed situations, Nrf2 protein is ubiquitinated by Keap1-Cullin3 E3 ubiquitin ligase enzymes and subsequently degraded by the proteasomal machinery [4, 5]. Upon exposure to electrophiles or oxidative stresses, the interaction between Nrf2 and that complex is interrupted, followed by decreased Nrf2 degradation and increased its translocation into the nucleus where heterodimerized with the small MAF (musculoaponeurotic fibrosarcoma) proteins (MafF, MafK, and MafG) (Fig. 1). These proteins are necessary for the Nrf2-related upregulation of antioxidant response element (ARE)-dependent target genes [5]. In response to the oxidative stress, about 200 cytoprotective genes are regulated by Nrf2 [9–12]. There are a lot of synthetic or plant-derived chemopreventive compounds, which exert their cancer-preventive roles by involving Nrf2-related defense responses [13–18]. Transient activation of Nrf2 is beneficial in countering carcinogens and mutagens and has protective roles versus tumor initiation in normal cells [19–22]. Howevere, in several of pathological conditions, comprising inflammation and cancer, some of the changes, such as somatic mutations in Nrf2,Keap1,and Cul3, Keap1 modification by metabolic mediators, epigenetically silencing of Keap1, Nrf2 transcriptional activation via oncogene-mediated signaling, and unusual accumulation of the proteins that disrupting the Keap1-Nrf2 interactions, lead to prolonged activation of Nrf2 [23–29]. Several studies have provided evidence that preventing the permanent activity of Nrf2 by its inhibitors renders cancer cells susceptible to apoptosis and enhances the efficacy of chemotherapeutics [30–33]. Therefore, Nrf2 is involved not only in biological defense against carcinogenesis and cancer development but also in cancer development and resistance to chemo-and radiotherapy. In the present review, we describe Nrf2 activators and inhibitors to contribute to obtaining an optimal balance between the tumor-preventive or -promoting activities of Nrf2 (Table 1).

**Table 1:** The selected activators and inhibitors of Nrf2 and their mechanisms of action

	Compound	Type	Mechanism of action	Reference
Nrf2 activators	SFN	Isothiocyanate	Electrophilic modification of Keap1-Cys-151	[87]
	OPZ	Organosulfur compound	Electrophilic modification of Keap1-Cys-151	[107]
	EGCG	Catechin	Oxidizing the cysteine thiols of Keap1	[110]
	DMF	Fumaric acid ester	Electrophilic modification of Keap1-Cys-151	[127]
	DATS	Isothiocyanate	Modification of Keap1-Cys-288	[141]
	CUR	Stilbene	Electrophilic modification of Keap1-Cys-151	[159]
	CDDO	Synthetic triterpenoids	Electrophilic modification of Keap1-Cys-151	[19]
	API	Plant flavone	Epigenetic modifications of Nrf2	[179]
	RES	(E)-Stilbene derivate	Electrophilic modification of Keap1-Cys-151	[188]
Nrf2 inhibitors	BRU	Triterpene lactone compound	Stimulation of Nrf2 poly-ubiquitination	[194]
	LUT	Plant flavone	Nrf2 mRNA degradation, Reduction of Nrf2 binding to AREs	[198]
	TRG	Coffee-derived alkaloid	Prevention of nuclear translocation of NRF2	[201]
	AA	Natural vitamin	Electrophilic modification of Keap1-Cys-151	[204]
	RA	Metabolite of vitamin A	Prevention of nuclear translocation of NRF2	[210]
	CHR	Plant flavone	Prevention of nuclear translocation of NRF2	[219]

*AA* Ascorbic acid; *API* Apigenin; *BRU* Brusatol; *CDDO* 2-cyano-3,12-dioxooleana-1,9(11)-dien-28-oic acid; *CHR* Chrysin; *CUR* Curcumin; *DATS* Diallyl trisulfide; *DMF* Dimethylfumarate; *EGCG* Epigallocatechin-3-gallate; *LUT* Luteolin; *OPZ* Oltipraz; *RA* Retinoic Acid; *RES* Resveratrol; *SFN* Sulforaphane; *TRG* Trigonelline

## Molecular mechanisms of Nrf2 regulation in cancer

### Somatic mutations in Keap1, Nrf2 and Cullin-3

**Fig. 2:**
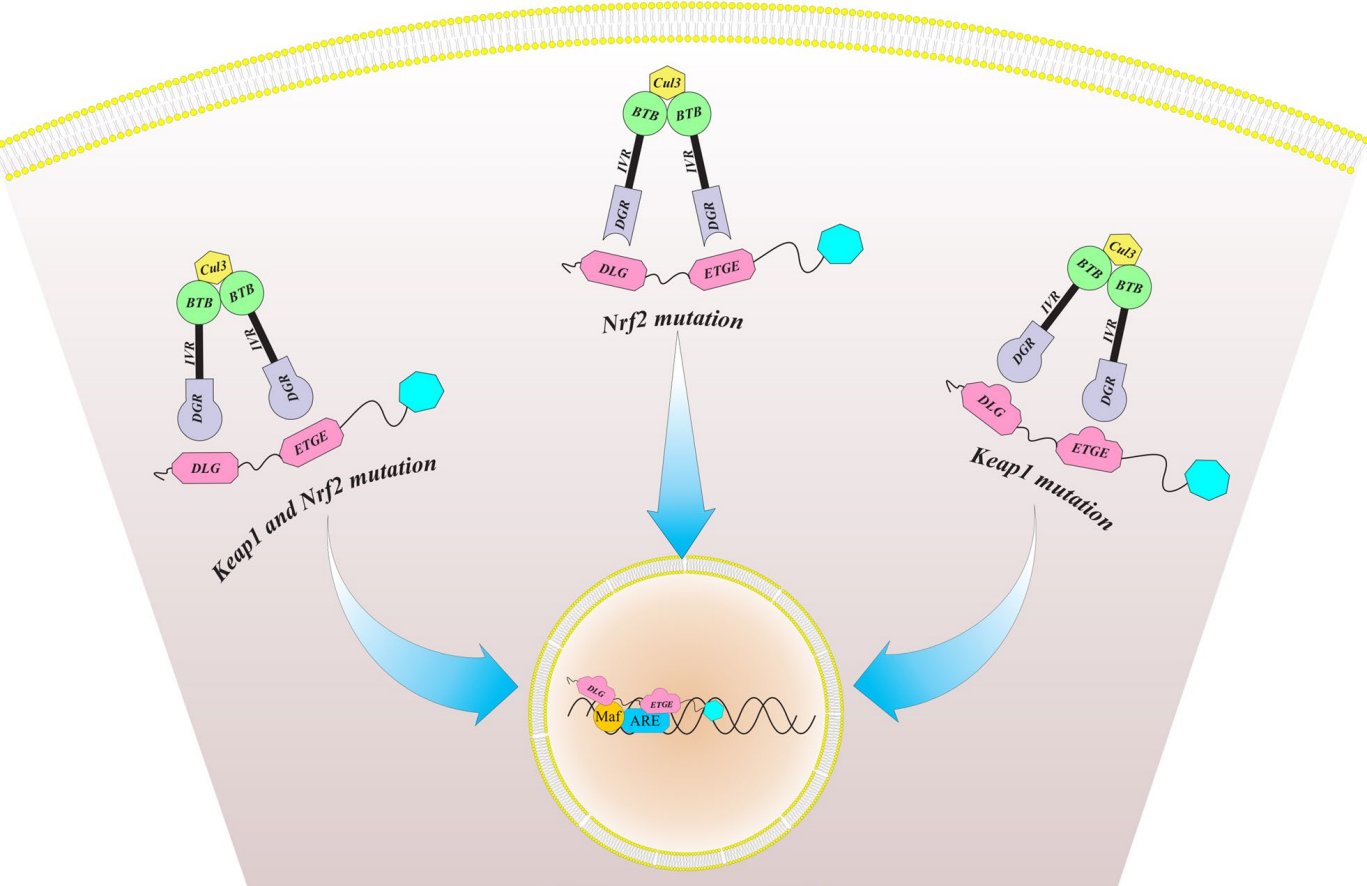
Somatic mutations of Keap1 and/or Nrf2 can result in nuclear accumulation of Nrf2

Initially, somatic mutations of Keap1 were recognized in lung tumor tissues and cell lines [34], which are the second most common and significant genetic modifications in lung cancer [35]. However, Keap1 mutations have also been reported in other human cancers, for example, ovary (19%), gastric (11%), liver (9%), colon (8%), prostate (8%), and breast cancer (2%) [36–38]. These mutations were reported in several Keap1 domains, which result in inactivation of Keap1 and accumulation of Nrf2 in the nucleus of cancer cells [39–42]. Besides the Keap1 mutations, gain of function mutations of Nrf2 have also been identified in human cancers for example esophageal carcinoma, lung, head and neck cancer [23, 27]. Interestingly, almost all Nrf2 mutations occur specifically within either the ETGE (57%) or DLG (43%) motifs [24]. When the mutations occur at the ETGE motif, the high-affinity interaction between the keap1 and Nrf2 is destroyed [26], while DLG motif mutations lead to destruction of low-affinity interaction [27] (Fig. 2). Recently, Ooiet al. [43] identified somatic mutations of Cul3 in sporadic papillary renal cell carcinoma type-2 (PRCC2). They showed that Cul3 mutation can be the trigger of Nrf2 activation in some of the sporadic PRCC2.

### Epigenetic silencing of Keap1

Epigenetic changes are the most common causes of Keap1 gene silencing in solid tumors and are obviously involved in the complicated regulation of the Keap1-Nrf2 system [44]. Multiple studies have indicated that epigenetic alterations in the Keap1 gene donate a growth advantage to cancer cells and are correlated with poor clinical prognosis in cancer patients [23, 45, 46]. The promoter hypermethylation of Keap1 that can lead to reduction of Keap1 expression and Nrf2 accumulation in the nucleus has been identified in malignant glioma [47], lung [48, 49], prostate [38], colorectal [50], gastric [51] and breast cancers [52]. Besides the effects of hypermethylation, the Keap1 expression can be affected by several microRNAs that act as oncogenes or tumor suppressor-microRNAs in tumor cells [53]. For example, *miR-7,miR-200a* and *miR-141* that are overexpressed in neuroblastoma, breast and ovarian cancer, respectively, down-regulate Keap1 expression through binding to the 3′-untranslated region (3′-UTR) of Keap1 mRNA [54–56], while inhibition of miR-200a results in up-regulation of Keap1 and subsequently reduction in Nrf2 activation [54].

### The role of proteins that disrupting the Nrf2-Keap1 interactions

p21, an inhibitor of cyclin-dependent kinase, is one of the non-electrophilic activators of Nrf2 that has been reported to relate to the DLG motif of Nrf2. Indeed, p21 disrupts two-site binding of Nrf2 to the Keap1 and results in stabilization of Nrf2 protein. Therefore, in the presence of p21, the expression of Nrf2 and its cytoprotective genes is elevated, which followed by promoted cell survival in response to oxidative stress [57, 58].

Another protein involved in the disruption of Keap1-Nrf2 interactions is p62, which links to the autophagy-mediated degradation and contains a Keap1-interacting region (KIR) domain. Interestingly, the STGE (Ser-Thr-Gly-Glu) motif in KIR domain is similar to the Nrf2 ETGE motif and therefore is responsible for the direct interaction between Keap1 and p62 proteins [59–61]. It has been suggested that STGE-binding motif in p62 is bound to the Kelch domain of Keap1 with lower affinity than ETGE motif [62]. However, serine phosphorylation (S351) of the p62 STGE motif can remarkably increase this affinity and facilitate p62-dependent autophagic degradation of Keap1 which leading to subsequent activation of Nrf2 [63]. Significantly, the unusual accumulation of p62 has been identified in certain cancers such as hepatocellular carcinoma [64], lung [65, 66], gastric [67, 68], breast [69, 70], and colon cancers [67, 71], which might increase the malignant behavior of these tumors through improving Nrf2 activity.

### Oncogene signaling mediated-Nrf2 upregulation

Although the Nrf2 protein level primarily is regulated by the mutation/degradation process, a different study has been conducted on the control of the Nrf2 gene transcription. DeNicola et al. [72] showed that the transcriptional start site of Nrf2 has Jun and Myc binding sites and therefore, the expression of Nrf2 and its downstream genes can be increased remarkably by activating the oncogenic alleles of C-MYC, BRAF, and KRAS (C-MYC^ERT12^, BRAF^V619E^, and KRAS^G12D^) which followed by more reduction in the intracellular redox environment. In another study, promoter analysis of Nrf2 showed that in regulatory region in exon 1 of Nrf2, a 2-O-tetradecanoylphorbol-13-acetate (TPA) response element (TRE) was activated by KRAS in human non-small cell lung cancer (NSCLC) cells [73]. The oncogenic KRAS can induce antioxidant program through MAPK-mediated Nrf2 activation in pancreatic cancer. Furthermore, KRAS silencing or obstruction of MAP kinase signaling pathway efficiently decrease Nrf2 level and elevate ROS formation [74].

## Nrf2 activators

**Fig. 3:**
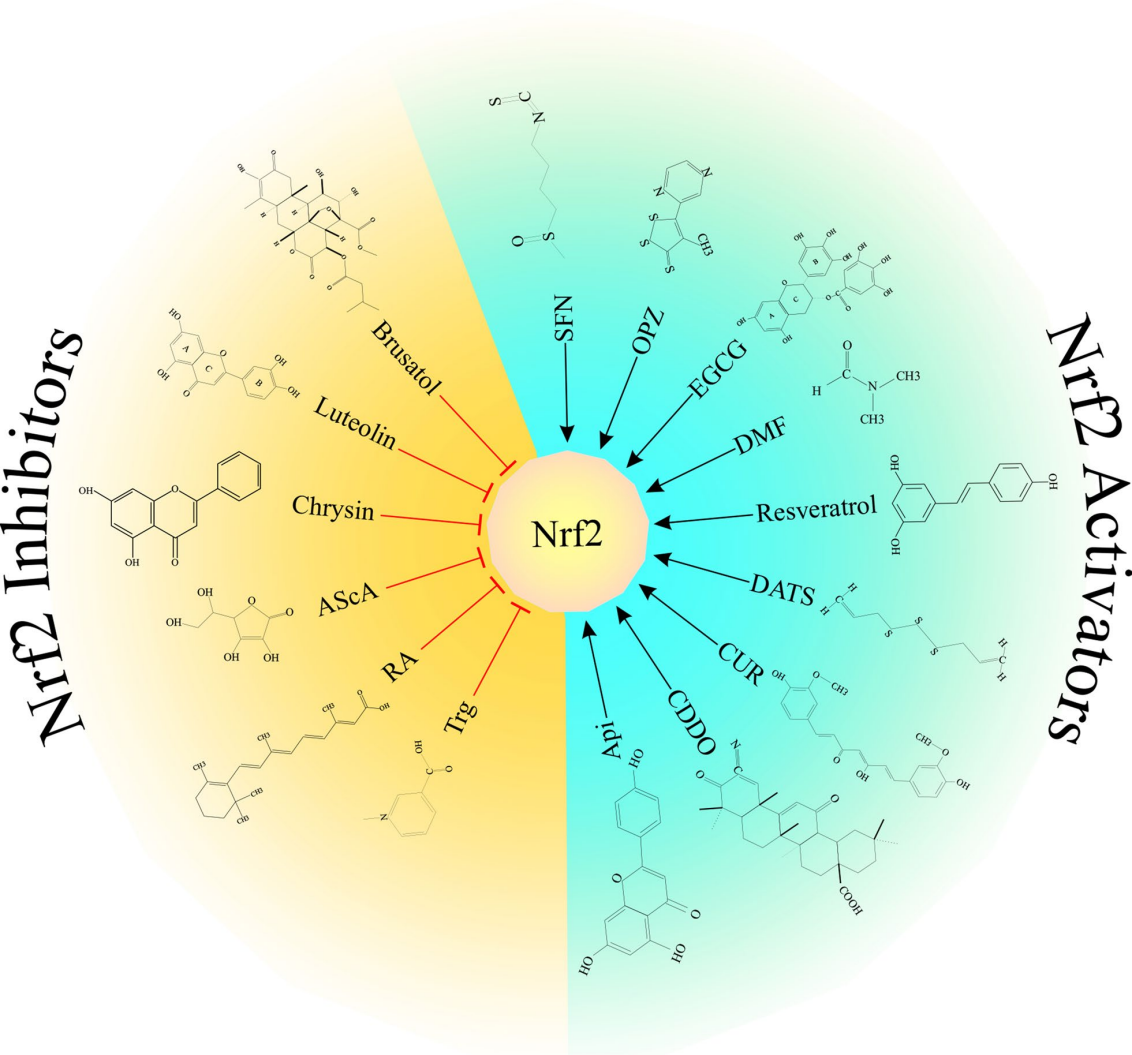
The common activators and inhibitors of Nrf2

There are many synthetic or extracted substances that function as Nrf2 activators (Fig. 3), which frequently are extracted from plants. Some examples of natural Nrf2 activators include curcumin, sulforaphane (SF), kahweol, resveratrol, garlic oganosulfur compounds, zerumbone, epigallocatechin-3-gallate, carnosol, cinnamonyl-based compounds, lycopene, and cafestol [75–77]. Magesh et al. [78] have categorized about 90 kinds of these synthetic or natural activators of Nrf2 in several groups: (1) isothiocyanates and sulfoxythiocarbamates; (2) oxidizable phenols and quinones; (3) Michael acceptors; (4) vicinal dimercaptans; (5) trivalent arsenicals; (6) dithiolethiones and diallyl sulfides; (7) heavy metals and metal complexes; (8) miscellaneous inducers; (9) selenium-based compounds; (10) polyenes; and (11) hydroxyl peroxides. By inducing the Nrf2-mediated defense response, these chemopreventive agents can activate the antioxidants, phase II detoxification factors, and transducers, and protect the cells from carcinogenic exposure [23].

### Sulforaphane [1-isothiocyanato-4-(methylsulfonyl)-butane] (SFN)

SFN is an effective dietary isothiocyanate, which found in cruciferous plants like Brussels sprouts and broccoli. SFN has anti-genotoxicity, anti-cancer and antioxidant activity as well as chemotherapeutic effect [79–81]. Besides enhancing cellular capacity in defense against oxidants, and electrophiles, it has been shown that sulforaphane is able to provoke apoptosis and reduce angiogenesis and cell cycle progression [82–84]. Interesting preclinical investigations show that sulforaphane prevents mice from forming carcinogen-mediated mammary carcinogenesis, lung, and gastric cancer, as well as colonic crypt foci [7, 85]. Previously, SFN was recognized as the most powerful activator of NQO1, but later it has been proved that the upregulation of NQO1 by SFN, indeed is regulated by the Nrf2-Keap1 signaling [86]. According to the in vivo experiments, site-directed mutagenesis and mass spectrometry analysis, it was evidenced that SFN can directly modify critical Keap1 cysteine 151 which followed by activation of the Nrf2-Keap1-ARE signaling [87, 88]. Thiols from Keap1 Kelch domain and isothiocyanate from SFN are covalently bound together which lead to the releasing of Nrf2 from Keap1 and finally inducing phase II metabolic enzymes [89, 90]. In another study, Kobayashi et al. categorized SFN as class 1 ARE inducers. Moreover, they found that the induction of ARE-regulated genes in zebrafish via sulforaphane is highly dependent on keap1 Cys151 [88]. It has been shown that SFN has a protective role against cancer development in different kinds of transgenic and carcinogen-induced tumor models (reviewed [91, 92]). In an animal study, Kalpana et al. revealed the inhibitory effect of SFN on benzo(a)pyrene (B(a)P)-induced lung cancer in the mouse, with emphasis on the effect of SFN on Nrf2 signal pathway [93]. Besides, in some clinical trial studies, the effect of SFN, as an Nrf2 activator, was evaluated in patients with colon, prostate, breast, and pancreatic cancers [79–82]. SFN can concomitantly upregulate Nrf2 and its downstream target genes, including HO1, NQOs, GSTs, and UGTs, rapidly in less than 30 min [94]. Another study demonstrated that sulforaphane epigenetically restored Nrf2 mRNA expression through the demethylation of its promoter CpGs in TRAMP-C1 and JB6 cells [95, 96].

### Oltipraz (4-methyl-5-[2-pyrazinyl]-1,2-dithiole- 3-thione) (OPZ)

OPZ, also known as a dithiolthione substitute, is one of the synthetic Nrf2 inducers [97]. Previous studies showed that OPZ can induce a number of Phase I and II antioxidant enzymes, especially that ones that increase glutathione levels, such as glucose-6-phosphate dehydrogenase, glutathione S-transferase (GST) and glutathione reductase [98–101]. The induction of GST and other cytoprotective enzymes has been termed the “antioxidant response”, which implies the possible impact of OPZ in cancer prevention [102, 103]. Moreover, OPZ is effective against many different kinds of common carcinogens such as 2-amino-1-methyl-6- phenylimidazo [4,5-b] pyridine (PhIP), aflatoxin B1 (AFB1) and benzo[a]pyrene (B[a]P) [104]. In another study, Sharma et al. demonstrated that oltipraz inhalation spray could inhibit B(a)P-mediated lung carcinoma in mice [105].

OPZ also may enhance the binding activity of Nrf2 to the ARE [100, 106]. Jia et al. [107] showed that dithiolethiones potentially have the ability to Keap1 cysteine modification. For example, 3H-1,2-dithiole-3-thione, which is structurally similar to OPZ, induces intermolecular disulfide cross-linking between two Keap1 monomers by targeting Cys273 and Cys288 [108]. Moreover, another study proved that Nrf2 is responsible for the chemopreventive effects of OPZ against bladder cancer [109].

### Epigallocatechin-3-gallate (EGCG)

EGCG is known to be a potent inducer of Nrf2 among several polyphenols found in green tea extract [110]. EGCG has anti-oxidative stress and anti-inflammatory activities through the downregulation of cyclooxygenase-2 and nitric oxide synthase [111]. According to the previous studies, EGCG has protective activity against experimentally induced prostate [112], fore-stomach [113], lung [114], skin [115], breast [116], and colon cancer [117]. Khan et al. [118] reported that EGCG could inhibit the cell adhesion function and downregulate the expression of matrix metalloproteinases that results in a reduction in angiogenesis, metastasis, and invasion of cancer cells. In addition to its ability to function as an anti-angiogenic agent, EGCG can induce apoptosis in numerous types of cancers by stabilizing the tumor suppressor p53 [119] and inactivating some transcription factors [119, 120].

It seems that EGCG by oxidizing or modifying Keap1 cysteine residues accelerates disassociation of Nrf2-Keap1 complex [121]. Moreover, EGCG is reported to activate Nrf2 via induction of upstream signalings, ERK and PI3K, which led to phosphorylation of Nrf2 serine/threonine residue in human mammary epithelial cells [122]. Other studies have described that EGCG inhibits the expression of Bach1, an Nrf2 competitor for binding to ARE sites (INrf2), in cultured A549 cells [123, 124].

### Dimethyl fumarate (tecfidera or DMF)

DMF, a methyl ester of fumaric acid (FA), is a chemical potent activator of Nrf2 [125]. The molecular mechanisms which DMF exerts its effects on Nrf2 are not understood completely but there are some suggested mechanisms. DMF, by activating the Nrf2-dependent anti-oxidant response pathway, stimulates the anti-inflammatory and cytoprotective responses [126, 127]. It also inhibits NF-κB-driven processes [128], and as an α, β carboxylic acid ester is able to bind to the thiol groups and modulate glutathione availability and production [127, 129, 130]. Ahuja et al. [131] demonstrated that DMF and DMF metabolite monomethylfumarate (MMF) activate the Nrf2 signaling through alkylation of Keap1 Cys residues and via induction of Bach1 exclusion from the nucleus. It has been demonstrated that Nrf2 activation by MMF is dose and time-dependent [132]. Furthermore, pre-treatment of neuronal cells with a low concentration of dimethylfumarate, by promoting cellular GSH, could protect them from oxidative glutamate toxicity [133, 134].

DMF-related activation of Nrf2 exerts cytoprotective actions in different cell types, for example, splenocytes, embryonic primary cortical cells, astrocytes, microglial cells, renal fibroblasts, and mesangial cells. Moreover, a growing body of evidence suggests that DMF suppresses proliferation, invasion, and angiogenesis and promotes apoptosis of various cancer cells [135–137]. An animal study showed that DMF has pro-apoptotic and anti-proliferative activities in melanoma cells and postpones progression and metastasis of melanoma [138].

### Diallyl trisulfide (DATS)

DATS is one of the well-known kinds of isothiocyanates (ITC) found in garlic oil and in a diversity of edible cruciferous vegetables, for example, cabbage, broccoli, and watercress [139]. In ex and in vivo experiments, DATS by modification of the Keap1, Cys288, is able to activate Nrf2 and promote NQO1 and HO-1 expression [140, 141].

In cardiomyoblast cells, the Nrf2 level and its nuclear accumulation, as well as the expression of its target antioxidant enzymes, were significantly higher in the cells treated with DATS. In addition, the Keap1 and GSK3β (enhancer of Nrf2 degradation) protein levels were significantly lower in those cells. Further analysis showed that DATS activates Nrf2 signaling by a MAPK-independent pathway which followed by suppression of hypoglycemia-induced apoptosis [142]. Moreover, it has been demonstrated that DATS can regulate drug metabolism by activation of Nrf2/ARE signaling. Because DATS-mediated detoxifying gene expression was detected in a wild-type mouse, but not in Nrf2 null mouse [143].

### Curcumin (CUR)

CUR, as a classical activator of Nrf2, is one of the well-investigated natural chemopreventive agents extracted from turmeric [144] (an Indian spice). CUR has a variety of therapeutic properties comprise of anti-oxidant [145], anti-inflammatory [146, 147] and anti-cancer activities [148, 149]. It also has been shown that CUR, via affecting the expression of AR, NQO1, GST, and HO-1, is able to activate a xenobiotic response in the cells [150, 151]. Subsequent analyses suggested that chemopreventive/therapeutic effects of CUR may be exerted by epigenetic alteration [152]. CUR, at lower concentrations, had demethylating effects on the promoter region of Nrf2, which resulted in elevated expressions of Nrf2 and its target genes [153]. Besides epigenetic modifications in Nrf2, curcumin may indirectly phosphorylate Nrf2 at serine- and/or threonine-rich regions and facilitate the nuclear transition of Nrf2. In addition, it can directly interact with sensor cysteine thiol(s) of Keap1 and diminish its inhibitory effect on Nrf2 [154]. Interestingly, CUR is able to play radiation and chemotherapy sensitizer role in some of the human cancers such as prostate [155], colorectal [156, 157] and ovarian cancer [158]. Some of the clinical trial studies indicated that CUR is quite safe and probably has therapeutic applicability in cancer treatment. Curcumin consumption for 3 months could improve the pre-cancerous lesions of patients with resected uterine cervical intraepithelial neoplasia, intestinal metaplasia, oral leukoplakia, and bladder cancer [159]. Despite the activatory effect of CUR on Nrf2 signaling, it is able to exert inhibitory effects on some other signalings, such as Notch1 [160], NF-kappa B [158] and mitochondrial signaling pathways [161].

### 2-cyano-3,12-dioxooleana-1,9(11)-dien-28-oic acid(CDDO)

CDDO is a synthetic triterpenoid analog, which applied in two types CDDO-Imidazolide (CDDO-Im) and CDDO-methyl ester (CDDO-Me), and conjugated with electron-withdrawing groups by covalent connection. At low concentrations (in picomolar range), CDDO-Im and CDDO-Me are the most effective known Nrf2 activators that have shown anti-inflammatory, pro-apoptotic, anti-proliferative and cytoprotective properties [162, 163]. Cleasby et al. [19] indicated that CDDO-Im can disrupt the BTB-Cul3 interface via covalent interaction with reactive cysteine 151 in the BTB domain of Keap1. Besides Keap1, CDDO-Im interacts with several targets such as PTEN, JAK1/STAT3, ErbB2, NF-kB, PPAR, mTOR and results in alterations in down-stream events [164, 165]. Yates et al. [163] showed that CDDO-Im may be an effective chemopreventive agent against cancers-mediated by electrophilic carcinogens or -associated with obesity. Another study showed chemopreventive potency of CDDO-Im against aflatoxin-induced hepatic tumorigenesis in an Nrf2-dependent manner [17]. Moreover, a knockout mouse study showed that CDDO-Me selectively induces the Nrf2/Keap1 signaling [166]. Significant alterations in the expression of 43 proteins were detected in the wild-type mice following intraperitoneal injection of CDDO-Me. However, among these altered proteins, only two proteins were similarly affected in Nrf2 null mice, indicating that almost all of these protein alterations were mediated through the Nrf2/Keap1 system. High doses of CDDO-Me (micromolar range) can suppress cancer cell proliferation and provoke apoptosis and cancer cell death in various tumor types [167, 168]. Furthermore, CDDO-Me has a higher potential to reduce lung tumor size in the mouse model in compare with CDDO-Im. Despite less efficacy in this lung cancer model, CDDO-Im has an effective role in the prevention and treatment of many other tumors including breast [169], prostate [170], and liver carcinogenesis [17].

### Apigenin (4,5,7-trihydroxyavone (Api))

Api is a kind of flavonoids that plentifully exists in plant-derived beverages and vegetables including orange, onion, parsley, tea, wheat sprouts and chamomile [171]. According to the previous studies Api has different pharmacological properties, for example, antivirus [172], anti-inflammatory [173], anti-oxidant [174] and anticancer activity [175]. In addition, Api, because of low bioactivity and slow pharmacokinetics, effectively accumulates in cells/tissues [176, 177]. Api potently increases the transcription of Nrf2 and leads to the elevated level of phase II detoxification proteins in t-BHP (tert-Butyl hydroperoxide)-treated ARPE cells (Retinal pigment epithelium cells). It is noteworthy that the endogenous mRNA and protein expressions of Nrf2 and its downstream gene, hemeoxygenase-1 (HO-1), considerably are elevated by Api, which followed by cellular protection against oxidative condition [178–180]. While knock down or knock out of the Nrf2 by CRISPER/Cas9 system or specific shRNA lead to decreasing the protective effects of Api in oxidative stress conditions [178]. Frequently, it was reported that Api by CpG site demethylation along with attenuated activities of DNA methyltransferase and histone deacetylases capable to restore the silenced status of Nrf2 in skin epidermal JB6 P + cell line [179]. Nevertheless, in contrast with the previous study, it has been reported that Api, via down-regulating of PI3K/Akt pathway, diminishes the expression of Nrf2 at both protein and mRNA levels which leads to a reduced expression of Nrf2-target genes in BEL-7402 cells (human hepatocellular carcinoma cells). Moreover, apigenin, in combination with chrysin, directly inhibits the PI3K/Akt pathway, which is associated with the survival of cancer cells [181–183].

### Resveratrol (3,5,4-trihydroxystilbene (RES))

Resveratrol, a naturally non-flavonoid polyphenol compound, is found in various food and plants for example: cranberry, mulberry, peanut, and the skin of red grape with different concentrations [184, 185]. Various environmental stress and stimulators, for examples excessive sunlight, UV irradiation, microbial and fungal infection, and mechanical injury, are capable of paving the way for producing the resveratrol by many plants species [184, 186]. Originally, there are two isomeric forms of resveratrol, trans-resveratrol and cis-resveratrol. Although both isomers are biologically active, the early one is more stable isomer and serves as a dominant form in the vast majority of resveratrol’s biological functions [186]. Numerous in vitro and in vivo studies have been illustrated that resveratrol occupies an axial role in modulating the signaling pathways associated with cellular growth and division, apoptosis, angiogenesis, invasion, and metastasis. Furthermore, resveratrol possesses anti-diabetic, anticancer, antioxidant, and anti-inflammatory effects [184–187]. In breast cancer, resveratrol through increasing the expression of Nrf2 and UGT1A8, an enzyme that can metabolize the catechol estrogen, contributes to the degradation of catechol estrogen [188, 189]. Zhang et al. found that resveratrol were capable of elevating the expression levels of Nrf2, HO-1 and reducing the level of ROS production and Keap1. Additionally, they reported that cell treatment with resveratrol, suppressed cell proliferation and Bcl-2 protein expression and stimulated expression of Bax protein and apoptosis [190]. According to the previous studies, it is remarkable to note that resveratrol is capable of activating the Nrf2/ARE signaling pathway and exerts antioxidant protective effects by regulating the expression of phase II detoxification enzymes [184, 185, 188].

## Nrf2 inhibitors

In contrast with the several agents that function as Nrf2 inducers, very few molecular components have been recognized as Nrf2 inhibitors. Since Nrf2 has multifaceted roles in cancer cells, Nrf2 inhibitors can be applied as anticancer agents [27, 77, 191]. Indirectly, Nrf2 inhibitors down-regulate drug detoxifying and eliminating enzymes and sensitize cancer cells to chemotherapeutics [76, 176]. According to the Nrf2 deactivation mechanisms and their potential applications in cancer treatment, several small molecules have been characterized as Nrf2 pathway inhibitors (Fig. 3).

### Brusatol (BRU)

Brusatol is a quassinoid which is extracted from Brucea Javanica (Simaroubaceae), an evergreen shrub grown in Northern Australia and Southeast Asia [27]. Initially, it was recognized as an anticancer agent against leukemia [192]. Brusatol changes the Nrf2 protein levels without changing in the Keap1 level [193]. Moreover, brusatol sensitizes various types of cancer cells, including HeLa cells, MDA-MB-231, and A549 cell lines, to some chemotherapeutics such as 5-fluorouracil, carboplatin, paclitaxel as well as etoposide [193]. This Nrf2 inhibitor also reduces tumor burden and improves survival in the A549 xenograft mice model [194]. However, there are several obstacles in the application of the brusatol as a therapeutic agent including toxicity, drug delivery and its effect reversibility that is necessary to be resolved [176]. Although it has been indicated that brusatol has transient and rapid post-transcriptional inhibitory effect on Nrf2 [195], the specificity and precise anticancer mechanism of brusatol have not yet been fully understood.

### Luteolin (3′,4′,5,7-tetrahydroxyflavone (LUT))

Luteolin is a natural polyphenolic flavonoid which obtained from various kinds of plants for example broccoli, celery, parsley, perilla leaf, and peppers, and characterized as one of the Nrf2 inhibitors [193, 196]. Luteolin has a wide range of biological effects such as antibacterial, antioxidant, anti-inflammatory and anticancer as well as cytoprotective activities [197]. Luteolin considerably enhances the anticancer efficiency of chemotherapeutic drugs, such as bleomycin, oxaliplatin, and doxorubicin, on A549 cell lines. When applying as an Nrf2 inhibitor, Luteolin reverses the sensitivity of colorectal cancer cells to the chemotherapy agents [198]. Additionally, luteolin is capable to suppress the cell cycle promotion and also acts as an apoptosis-inducer and anti-proliferative agent in several cancers, for example, gastric, prostate and pancreatic cancer, hepatoma, melanoma, leukemia, and epidermoid carcinoma [196]. It should be noted that luteolin in some studies has been categorized as an Nrf2 activator, on the other [180, 199, 200].

### Trigonelline (TRG)

Trigonelline, a heterocyclic compound, is widely existing in plants, coffee and fenugreek seed that in comparison with chemicals is less toxic to humans [201]. Recently it has been identified that Trg has anti-diabetic, hypocholesterolemic, antimigraine, anticancer, as well as, Nrf2-inhibitory effects [196, 201] which blocks the Nrf2-dependent expression of proteasomal genes [193]. In another study, Trg reversed resistance to ferroptotic cell death in head and neck cancer by blocking the Nrf2 pathway, both in vitro and in vivo [202]. It has been recognized that using a combination of etoposide and trigonelline can lead to the enhancement of anticancer efficacy of etoposide and reduction in tumor size [196], especially in the tumors have high-level activity of Nrf2 [193].

### Ascorbic acid (vitamin C, L-ascorbic acid, AscA, AA)

Ascorbic acid which generally known as an antioxidant agent [176], suppresses the Nrf2/DNA complex [78] and through inhibition of the nucleus translocation of Nrf2, reduces the cellular level of peroxides [176]. Additionally, AA, because of abilities in the hydrogen peroxide generation, is categorized as a pro-oxidant which sensitizes tumor cells to the therapeutics, but sometimes has been observed the opposite effects [176, 203]. Based on S.R. Kim et al. [204] results, AA through involving the Cys151Ser in the Keap1, leading to activation of PI3K/Nrf-2 and finally inducing the HO-1 which has antioxidant effects by increasing the level of reduced glutathione [205]. In another study,Vineetha RC et al. [206] reported AA by elevating the level of oxidative stress leading to the up regulation of Nrf2 and Bcl2 expression.

### Retinoic acid (RA)

Retinoic Acid (RA), also known as All-trans-retinoic acid (ATRA), is a metabolite of vitamin A [207]. RA by ARE-inducing elements, for example, tBHQ, decreases the capability of Nrf2 to mediate the induction of ARE-regulated genes in both in vivo and ex vivo conditions [78]. RA has biological functions such as regulating cell differentiation, proliferation, and morphogenesis. RA by stimulation of cellular differentiation and suppression of cell growth inhibits tumorigenesis [208] and also is able to enhance the apoptosis [209] which this property may contribute to the anticancer role of RA [210]. RA exerts its effects by retinoid X receptors (RXR-a, b, and g) and retinoic acid receptors (RAR-a, b, and g), which are specific nuclear receptors. RARs, RXRs and/or other hormone receptors of the nucleus, create heterodimers and act as transcriptional regulators [210], which through binding to the transcription factors like Nrf2, prevent the binding of this transcription factors to the ARE [176]. Furthermore, the interaction between RAR/RXR heterodimers and other transcription factors like estrogen receptor, AP-1 and NF-B leads to the RA activation or repression and subsequently gene expression variations [211]. Although RA has been identified as Nrf2 inhibitor, in some studies it has been reported as an Nrf2 activator [23]. Therefore, prior to using RA as an anticancer agent, it is necessary to carry out additional studies on the specificity and mechanism of its actions.

### Chrysin (5,7-dihydroxy-2-phenyl-4H-chromen-4-one (CHR))

Chrysin, a natural flavonoid, is found in many plant extracts including honey, propolis, mushroom, blue passion flower, vegetables, and fruits [212]. According to the literature reports, the most reliable pharmacological activities of chrysin are antioxidant, anti-inflammatory, anti-diabetic, hepatoprotective, neuroprotective, anti-aging, and anticancer effects [213–215]. In many studies, it has been reported that chrysin occupies an axial role in many biological process for example suppression the pro-inflammatory cytokines expression, down-regulation of nuclear factor kappa B (NF-kB), tumor necrosis factor α (TNF-α), and interleukin 1β (IL-1β), up-regulation of apoptotic pathways, and inhibition of angiogenesis and metastasis formation [216–218]. Gao and colleagues have been reported a higher level of Nrf2 expression in BEL-7402/ADM cells. They showed that chrysin, by quenching ERK and PI3K-Akt pathway, makes a contribution to inhibition of Nrf2 and its downstream target genes as well as AKR1B10, HO-1, and MRP5 expression [219]. Additionally, Zeng et al. [220] disclosed that chrysin treatment promotes the expression of osteogenesis genes in preosteoblast MC3T3-E1 cell lines by activation of ERK/MAPK signaling pathway. In another study, it has been revealed that chrysin reduces the mRNA expression of Nrf2, MRP1, NQO-1, and HO-1 in breast cancer MCF7 cell lines [221]. It is completely obvious that chrysin decrease both mRNA and protein expression levels of Nrf2, however, it should be emphasized that the effects of chrysin on the Nrf2-ARE signaling pathway appears to be cell type-specific, concentration dependent, and may vary depending on the nature of Nrf2 regulation.

## Conclusion

Since Nrf2 has paradoxical roles in cancer biology, it is essential to understand the molecular mechanisms leading to tumor suppressor or oncogenic effects of Nrf2. Furthermore, to pave the way for identifying therapeutic strategies based on Nrf2 signaling in malignancy treatment, it has to be considered when the specific Nrf2 inducer or inhibitor is appropriate. However, further studies should be conducted to find cancer targeting drug candidates with good pharmacodynamic/pharmacokinetic parameters for human cancer.

## Data Availability

The authors confirm that the data supporting the findings of this study is available within the article.

## Abbreviations

Nrf2: NF-E2-related factor 2
ROS: Reactive oxygen species
RNS: Reactive nitrogen species
bZIP: Basic-region leucine zipper
MAF: Musculoaponeurotic fibrosarcoma
SF: Sulforaphane
GST: Glutathione S-transferase
EGCG: Epigallocatechin-3-gallate
MMF: Metabolite monomethyl fumarate
